# Evaluating therapeutic plasma exchange and protease inhibitors as mechanisms to reduce soluble mesothelin

**DOI:** 10.1038/s41598-025-97952-x

**Published:** 2025-04-16

**Authors:** Katherine E. R. Smith, Jennifer R. Ayers-Ringler, Jacob J. Orme, Fabrice Lucien, Yohan Kim, Jeffrey L. Winters, Aaron S. Mansfield

**Affiliations:** 1 Medical Oncology, Mayo Clinic, 200 1st St SW, Rochester, MN 55905 USA; 2 Department of Urology, Mayo Clinic, Rochester, MN USA; 3 Department of Immunology, Mayo Clinic, Rochester, MN USA; 4 Division of Transfusion Medicine, Mayo Clinic, Rochester, MN USA

**Keywords:** Soluble mesothelin, Mesothelioma, Soluble oncogenic proteins, Therapeutic plasma exchange, Protease inhibitors, Predictive markers, Cancer therapeutic resistance

## Abstract

Cell surface mesothelin (MSLN) can be solubilized and released into the systemic circulation. The resulting soluble MSLN (sMSLN) may interfere with therapies targeting surface MSLN. We investigated the effects of sMSLN on anetumab, an antibody-based therapy against MSLN, anetumab ravtansine, an antibody drug conjugate, and mechanisms to decrease sMSLN. Whole blood samples were collected before and after one plasma volume of therapeutic plasma exchange (TPE). sMSLN levels were measured with ELISA assays in matched pre- and post-TPE plasma samples, and anetumab-immunoprecipitated samples. We also used protease inhibitors (PIs) as a mechanism to stabilize surface MSLN, then evaluated the cytotoxic effects of anetumab ravtansine. Our findings indicate that sMSLN sequesters and may impair the efficacy of this anti-MSLN antibody based on results showing that anetumab decreases the concentration of MSLN in plasma (*p* < 0.05) and reduced cytotoxicity of anetumab ravtansine in the presence of recombinant MSLN in cell lines, a surrogate for sMSLN. TPE consistently reduced sMSLN (*p* < 0.05) with an average decrease of 43.6% (15.4 ng/mL). Surface MSLN stabilization was inconsistently observed with PIs. Overall, sMSLN could represent a predictive biomarker for MSLN directed therapies. TPE may be more reliable than PIs to reduce sMSLN and ultimately restore sensitivity to these therapies in patients with high sMSLN.

**Supplementary Information:**

The online version contains supplementary material available at 10.1038/s41598-025-97952-x.

## Introduction

There are many efforts underway to target mesothelin (MSLN), a cell surface protein overexpressed by epithelioid mesothelioma, non-small cell lung cancer (NSCLC), gynecologic cancers (ovarian, endometrial, cervical, vulvar), pancreatic cancer, head and neck cancer, and esophageal cancer, while maintaining limited expression in normal tissues^1–4^. Drugs targeting MSLN, including immunotoxins, vaccines, antibody drug conjugates (ADCs), T cell receptor (TCR) fusion constructs, and chimeric antigen receptor T-cells (CAR-T) have yet to show durable responses in mesothelioma^5–10^. MSLN is cleaved by proteases and released from the cell surface as soluble MSLN (sMSLN), which may contribute to treatment resistance. Recently, a phase I/II trial of anetumab ravtansine, an anti-MSLN ADC, plus pembrolizumab, compared to pembrolizumab alone in mesothelioma found that higher sMSLN levels were associated with worse progression free survival (PFS) for patients treated with anetumab ravtansine.There was no difference in PFS based on sMSLN levels for the pembrolizumab control arm^10^. Prior studies have associated high sMSLN levels with higher risk mesothelioma, but these recent results suggest that high sMSLN levels may impair the efficacy of anti-MSLN ADCs^10^.

In addition to MSLN, other surface proteins, such as programmed death ligand 1 (PD-L1), lymphocyte activation gene 3 (LAG3), and B-cell maturation antigen (BCMA) can also be solubilized, and higher levels of these soluble biomarkers are associated with worse clinical outcomes^11–18^. In the case of soluble PD-L1 (sPD-L1), the proteases ADAM10 and ADAM17 cleave PD-L1, and inhibition of these proteases leads to reduced sPDL1 in cell lines^19^. For MSLN, multiple proteases from the ADAM, BACE, and MMP families are involved in cleaving MSLN from the cell surface, resulting in sMSLN, a 40 kDa protein^1,16,20^. sMSLN levels are elevated in 70% of patients with epithelioid mesothelioma, and higher levels are associated with higher tumor burden, increased metabolic activity, and a worse prognosis^16–18^. Circulating MSLN is known to interfere with some MSLN-directed therapies, including sMSLN impairing a MSLN-specific immunotoxin and MSLN positive extracellular vesicles (EVs) (MSLN + EVs) inhibiting a MSLN CAR-T product^6,21–23^. Soluble PD-L1, LAG3, and BCMA are also implicated in resistance to immune checkpoint inhibitors (ICIs) and CAR-T^11,14,15^. We hypothesized that sMSLN can sink antibody-based MSLN therapies as is seen with these other soluble oncogenic proteins.

Protease inhibition remains the principal strategy to stabilize surface MSLN with the goal of reducing sMSLN and MSLN + EV levels in studies on MSLN immunotoxins and CAR-T, respectively^6,22,23^. However, protease inhibitors (PIs) can have off-target effects and induce cytotoxicity, which could lead to more side effects for patients. Therapeutic plasma exchange (TPE) may represent a novel approach to decrease sMSLN and potentially improve the efficacy of MSLN therapies. Recently, we reported that TPE decreases sPD-L1, which led to a trial investigating if TPE in combination with radiation prior to ICIs could decrease sPD-L1 to restore anti-cancer immunity in patients with metastatic melanoma^11^ (NCT04581382). TPE is a routine procedure with indications in certain autoimmune disorders and hyperviscosity syndromes where the patient’s blood is removed, the plasma is separated out in a medical device, then the plasma is exchanged with replacement fluid^24^. The procedure reliably removes proteins in the plasma that are larger than albumin (65–70 kDa), but is known to remove smaller substances as well, as seen with sPD-L1 (35 kDa) and PD-L1-positive EVs (PD-L1 + EVs) (50–500 nm)^11,24^.

Given the ongoing interest in developing MSLN-directed therapies throughout oncology with multiple active clinical trials, we sought to (1) investigate if sMSLN neutralizes anetumab-based therapies, (2) assess if the addition of the PIs would improve the cytotoxicity of anetumab ravtansine, and (3) determine the feasibility of utilizing TPE to decrease sMSLN. Our results provide insight into improving the MSLN-directed therapies currently in development while also contributing to the broader understanding of how solubilized oncogenic proteins can compromise treatments against cell surface targets.

## Results

### Therapeutic plasma exchange

**Fig. 1:**
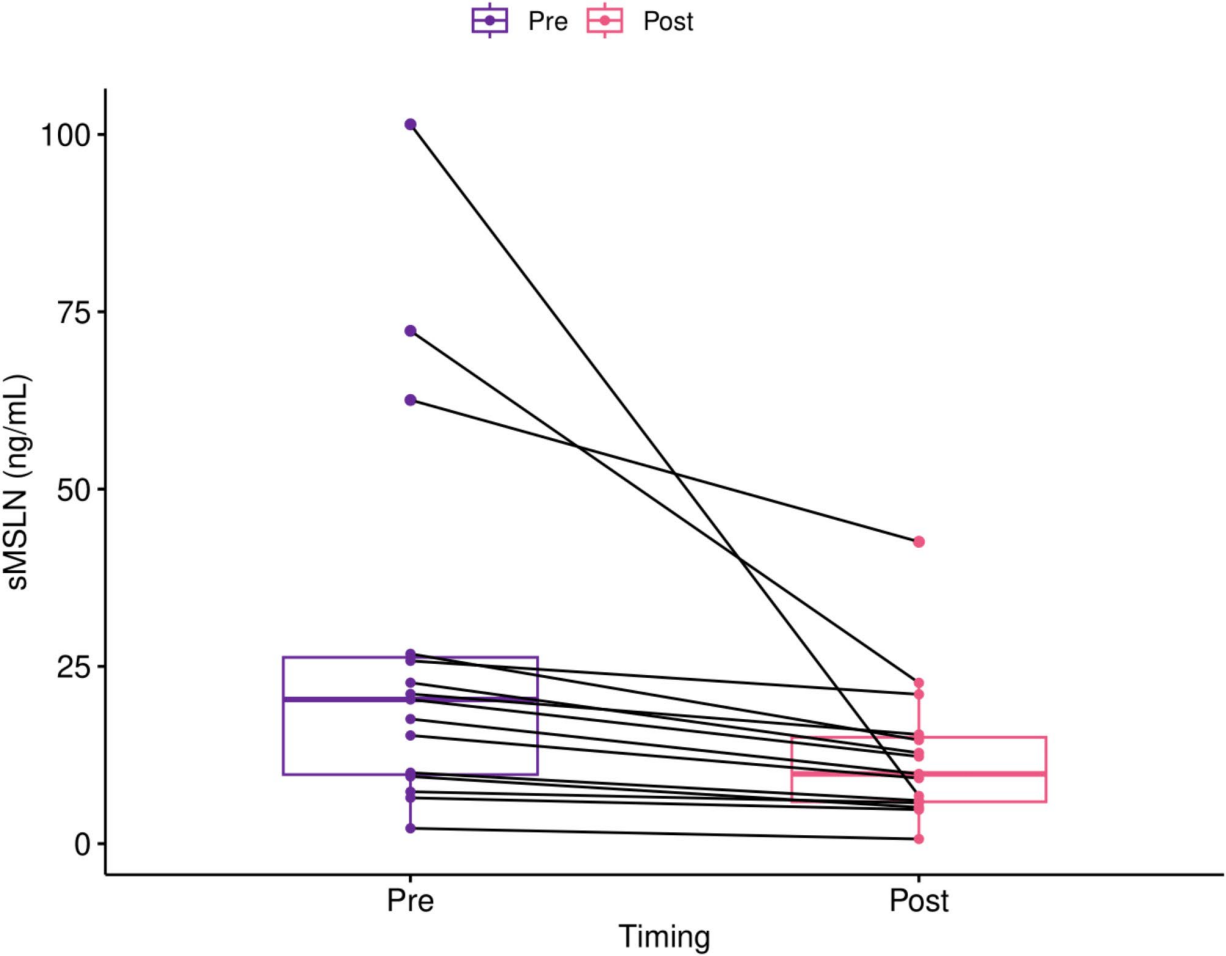
Matched Pre- and Post-Therapeutic Plasma Exchange Soluble Mesothelin Levels. Soluble mesothelin (sMSLN) levels were evaluated in 15 patients before and after one plasma volume of therapeutic plasma exchange (TPE). After TPE, sMSLN levels were consistently reduced (*p* = 0.031) with an average decrease of 43.6% or 15.4 ng/mL.

We evaluated pre- and post-TPE plasma samples from 15 patients undergoing routine TPE for various medical conditions, including central nervous system (CNS) demyelinating disorders (46.67%), paraneoplastic syndromes (13.33%), paraproteinemia (13.33%), Susac syndrome (13.33%), solid organ transplant rejection (6.67%), and immune encephalitis (6.67%) (Table 1). The CNS demyelinating disorders were either chronic inflammatory demyelinating polyradiculoneuropathy (CIDP), multiple sclerosis (MS), neuromyelitis optica (NMO), or myelitis. Seven patients were female and 8 were male. The mean age was 58 years old (ranging from 25 to 84 years old). Four patients (26.67%) were diagnosed with an active malignancy (melanoma, uterine neuroendocrine malignancy, non-Hodgkins’s lymphoma, and lymphoplasmacytic lymphoma) and three (20%) had a history of cancer (renal cancer carcinoma, anal cancer, and ovarian cancer). In the matched pre- and post-TPE samples, a one plasma volume TPE consistently reduced sMSLN (*p* = 0.031) with an average decrease of 43.6% or 15.4 ng/mL (Table 2, Fig. 1). sMSLN levels decreased for all patients regardless of their oncologic history.

**Table 1:** Patient characteristics.

Characteristics	Patient data (n = 15)
Age	58 (25–84)
Gender	Female—7 (46.67%)
	Male—8 (53.33%)
Cancer diagnosis	History—3 (20%)
	Active—4 (26.67%)
	None—8 (53.33%)
Indication for TPE	CNS demyelination—7 (46.67%)
	Paraneoplastic syndrome—2 (13.33%)
	Paraproteinemia—2 (13.33%)
	Susac syndrome—2 (13.33%)
	Transplant rejection—1 (6.67%)
	Immune encephalitis—1 (6.67%)
Exposure to steroids	Yes—7 (46.67%)
	No—8 (53.33%)
Hemoglobin prior to TPE, Mean	12.15 g/dL (8.1–15.5)
WBC prior to TPE, Mean	8.87 109/L (4.8–19.8)
Creatinine prior to TPE, Mean	1.03 mg/dL (0.61–1.86)

Categorical variables—n (%).

Continuous variables—mean (range).

TPE, therapeutic plasma exchange, WBC, white blood cell count.

**Table 2:** Soluble mesothelin levels pre- and post- therapeutic plasma exchange.

Patient	Pre-TPE	Post-TPE	% change
1	21.11	15.4	− 27.05
2	26.77	14.61	− 45.42
3	9.47	5.12	− 45.93
4	72.31	22.68	− 68.64
5	6.47	4.8	− 25.81
6	20.33	12.29	− 39.55
7	10.01	6.08	− 39.26
8	7.33	5.75	− 21.56
9	25.78	21.06	− 18.31
10	2.17	0.66	− 69.59
11	17.58	9.84	− 44.03
12	15.26	9.24	− 39.45
13	62.56	42.57	− 31.95
14	101.43	6.73	− 93.36
15	22.7	12.8	− 43.61

TPE, Therapeutic plasma exchange.

Measured in ng/mL.

### Anetumab immunoprecipitation

In two randomly selected plasma samples, which included a patient with a CNS demyelinating disorder and a patient with Susac syndrome, we found that the addition of anetumab-conjugated Dynabeads® significantly reduced the concentration of sMSLN in plasma samples as detected by a MSLN ELISA (*p* < 0.05). The first plasma sample had a baseline average sMSLN of 0.647 ng/mL, which decreased to an average of 0.229 ng/mL (64.5% decrease) with 12.5 µg/mL of anetumab, 0.352 ng/mL (45.6% decrease) with 25 µg/ml of anetumab, and 0.263 ng/mL (59.4% decrease) with 37.5 µg/mL of anetumab. The second plasma sample started with a baseline average sMSLN of 7.231 ng/mL which decreased to an average of 6.685 ng/mL (7.6% decrease) with 12.5 µg/mL of anetumab, 6.242 ng/mL (13.7% decrease) with 25 µg/mL of anetumab, and 6.703 ng/mL (7.3% decrease) with 37.5 µg/mL of anetumab. These results suggested to us that anetumab-conjugated Dynabeads repeatedly reduced sMSLN from plasma.

### Protease inhibitors and cytotoxicity

**Fig. 2:**
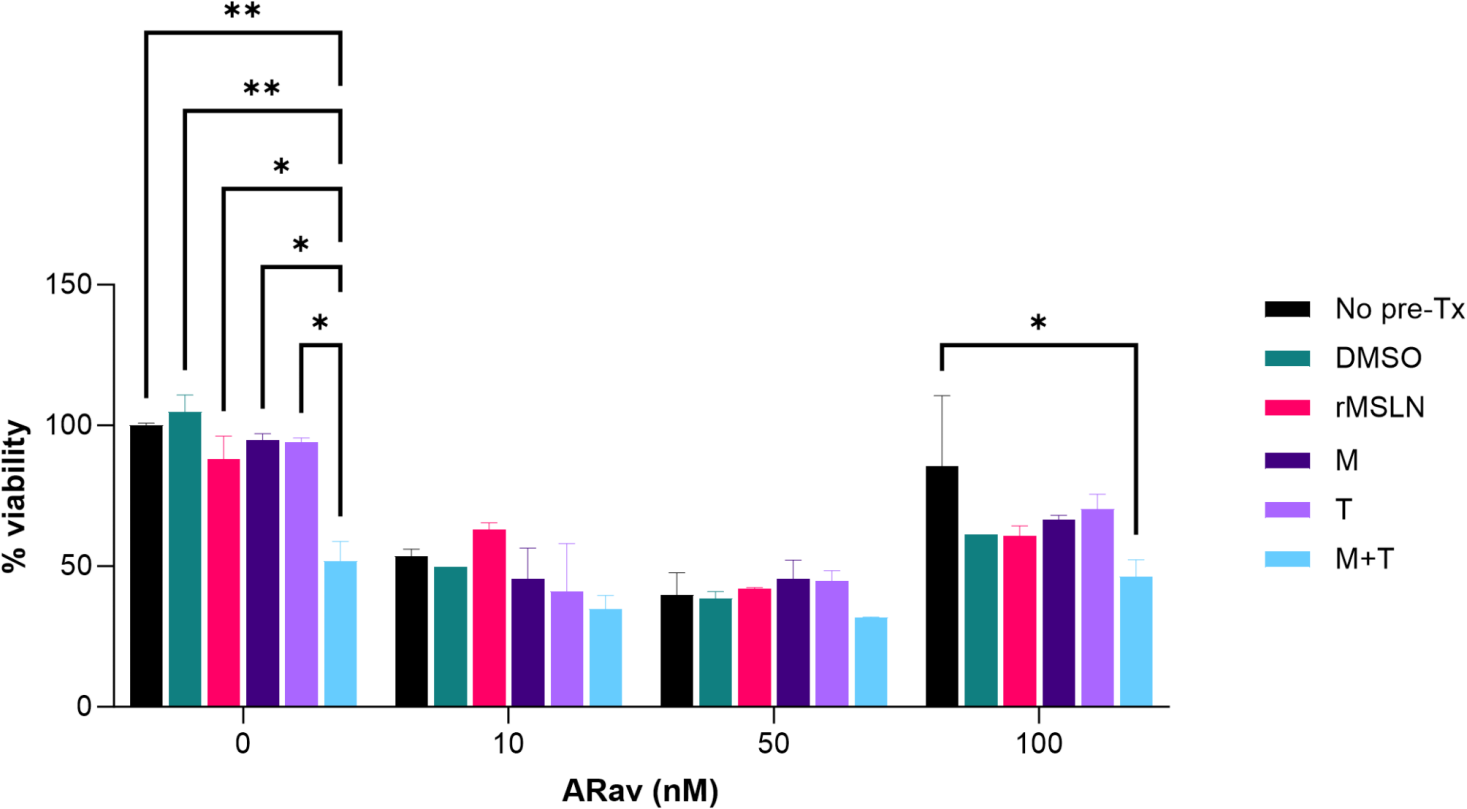
Cytotoxicity of Anetumab Ravtansine as Measured with Cell Viability. The effects of anetumab ravtansine at 0 nM, 10 nM, 50 nM, and 100 nM on mero-95 cell lines in the presence of no pretreatment (pre-Tx), DMSO control, recombinant MSLN (rMSLN), marimastat (M), TMI-1 (T), and a combination of both protease inhibitors (M + T). At baseline, the combination M + T resulted in decreased cell viability compared to the control (*p* = 0.0016), rMSLN (*p* = 0.0453), M (*p* = 0.012), T (*p* = 0.014). There was a trend of decreased viability at 10 nM, but this was not statistically significant. At 50 and 100 nM, M + T continued to reduce viability, which was significant at 100 nM. Otherwise, there were no significant differences between the groups at 10, 50, and 100 nM. See Supplemental Table 1 for more detailed results. ARav = anetumab ravtansine. * *p* ≤ 0.05, ** *p* ≤ 0.01.

**Fig. 3:**
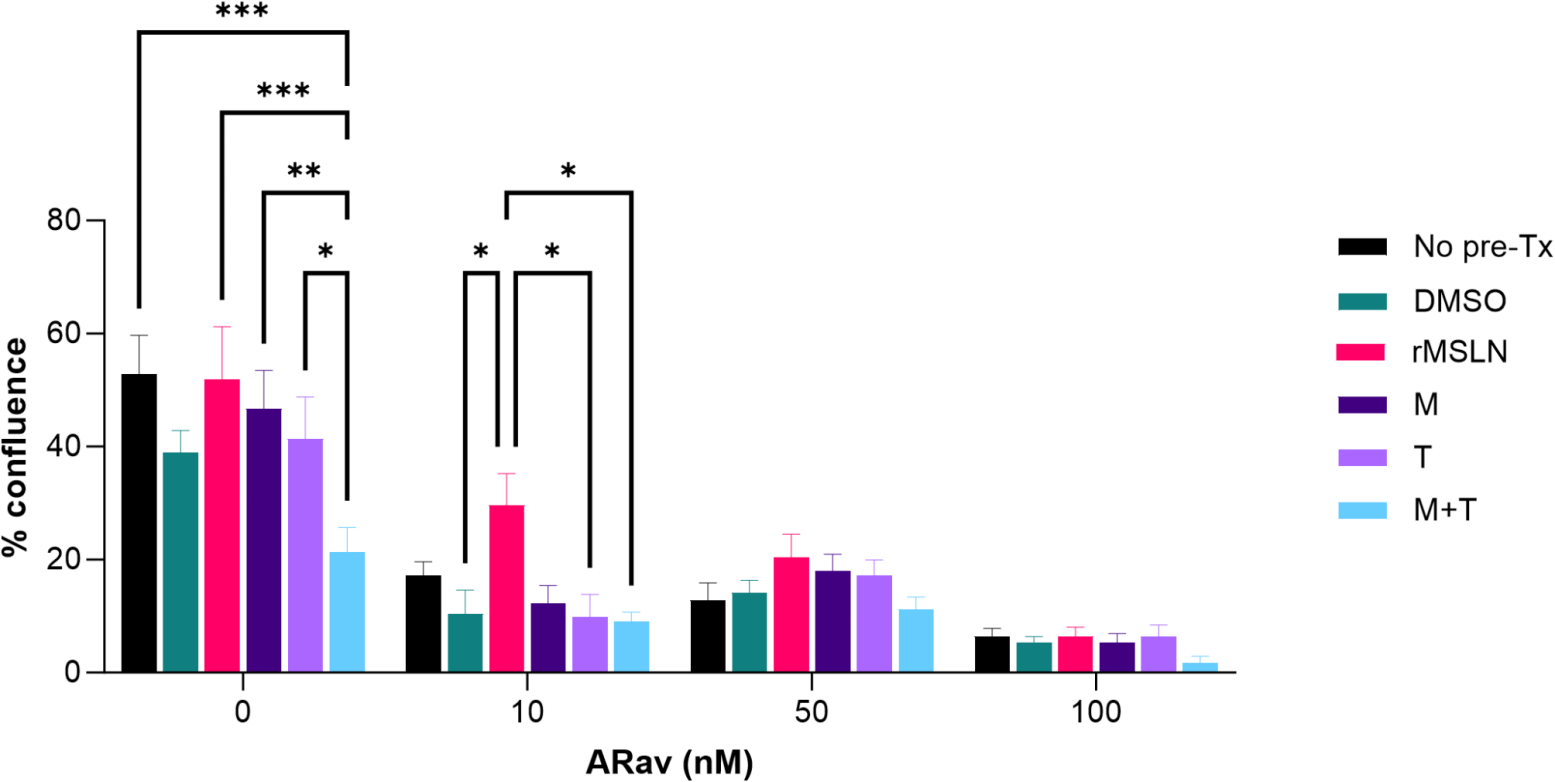
Cytotoxicity of Anetumab Ravtansine as Measured with Cell Confluence. The effects of anetumab ravtansine at 0 nM, 10 nM, 50 nM, and 100 nM on mero-95 cell lines in the presence of no pretreatment (no pre-tx), 1% DMSO control, recombinant MSLN (rMSLN), marimastat (M), TMI-1 (T), and a combination of both protease inhibitors (M + T). At baseline, the combination M + T resulted in decreased confluence compared to rMSLN (*p* = 0.0003), M (*p* = 0.0028), T (*p* = 0.0252). At 10 nM of anetumab ravtansine, the addition of rMSLN resulted in higher confluence or less cytotoxicity compared to control DMSO (*p* = 0.0348), T (*p* = 0.0289), M + T (*p* = 0.0206) but not M (*p* = 0.0679). This trend continued at 50 nM. At 100 h nM, there were no significant differences between the groups. See Supplemental Table 2 and Fig. 1 for more detailed results. ARav = anetumab ravtansine. * *p* ≤ 0.05, ** *p* ≤ 0.01.

Next, we evaluated the cytotoxicity of anetumab ravtansine at 0 nM, 10 nM, 50 nM, and 100 nM in the presence of (1) PIs to stabilize surface MSLN and (2) recombinant MSLN (rMSLN) as a surrogate for sMSLN (Figs. 2 and 3, Supplemental Tables 1 and 2, Supplemental Fig. 1). The protease inhibitors included marimastat (M), a broad-spectrum matrix metalloproteinase (MMP) inhibitor, TMI-1 (T), an inhibitor for ADAM17 and MMPs, and a combination of both (M + T). M and T were utilized in the initial studies that identified the proteases responsible for MSLN cleavage and were reported to reduce MSLN shedding^20^. Furthermore, we tested M and T on cell lines that express MSLN in preliminary experiments, which showed that both PIs reduced free MSLN in the medium at various concentrations.

At baseline, prior to the addition of anetumab ravtansine, there was no significant difference between a 1% DMSO control, M, and T on either cell viability or confluence. However, the combination of M + T resulted in decreased cell viability compared to no pretreatment (mean difference 48.25%, *p* = 0.0042), control DMSO (mean difference 53%, *p* = 0.0016), rMSLN (mean difference 36.2%, *p* = 0.0453), M (mean difference 43.1%, *p* = 0.012), T (mean difference 42.3%, *p* = 0.014). This trend continued for M + T at the other concentrations, and was significant at 100 nM (mean difference 39.6%, p = 0.026). Baseline M + T confluence also decreased compared to no pretreatment (mean difference 31.61%, *p* = 0.0002), rMSLN (mean difference 30.71%, *p* = 0.0003), M (mean difference 25.43%, *p* = 0.0029), T (mean difference 20.06%, *p* = 0.0252). These results suggest that M + T has a direct cytotoxic effect.

At 10 nM of anetumab ravtansine, the addition of rMSLN reduced confluence (mean difference 19.21%, *p* = 0.0348) but not viability (*p* = 0.927) compared to the DMSO control. This trend continued with 50 nM of anetumab ravtansine for confluence, but was not significant. Also at 10 nM anetumab ravtansine, the cells treated with rMSLN had higher percentage of confluence compared to T (mean difference 19.7%, *p* = 0.0289), and M + T (mean difference 20.58%, *p* = 0.0206) but not M (*p* = 0.0679), suggesting that the PIs may have stabilized surface MSLN to some degree, thus allowing anetumab ravtansine to be more effective, but this is difficult to put into context given that M + T may have direct cytotoxicity. At 100 nM of anetumab ravtansine, except for the reduced viability with M + T, there were no significant differences between the groups for either viability or confluence, which may be due to the high ADC concentration or low cell counts.

## Discussion

sMSLN can bind to and neutralize anti-MSLN antibodies in the plasma based on our results showing (1) anetumab added to plasma lowers sMSLN levels, and (2) anetumab ravtansine cytotoxicity is reduced in the presence of rMSLN. The recent clinical trial NCI ETCTN 10107 comparing anetumab ravtansine plus pembrolizumab to pembrolizumab alone in patients with mesothelioma found that higher sMSLN levels were associated with decreased PFS for the experimental arm only^10^. The differential findings for PFS based on sMSLN levels with anti-MSLN therapy compared to anti-PD1 therapy, in addition to the findings reported herein, indicate that sMSLN binds to and sequesters the MSLN antibody-based therapies before reaching the tumor surface^10^. Similar findings are observed for other MSLN-directed therapies, including immunotoxins and CAR-T products^6,10,22,23^. In mice treated with SS1P, a MSLN immunotoxin, sMSLN surrounding the tumor and in the blood blocked the cytotoxic effects of SS1P^21,22^. Additionally, MSLN + EVs can interfere with MSLN-directed CAR-Ts. When exosome release is inhibited with GW4869 and Nexinhib20, the activity of the MSLN-targeted CAR-Ts improved^6^. Further, studies of MSLN directed CAR-T and TCRs have found that the levels of sMSLN related peptides (SMRP) decreases with each therapy, which could be due to the therapies binding to SMRPs in the plasma since a decline in SMRP does not necessarily indicate response to therapy^5,25^. In addition to MSLN, other solubilized tumor surface proteins, including PD-L1, BCMA, and LAG3 are also implicated in treatment resistance^11,14,15^.

Since sMSLN can undermine the efficacy of MSLN-directed therapies, reduction of sMSLN may improve clinical responses. PIs are the most studied option for stabilizing both surface MSLN and PD-L1^6,19–21^. In pre-clinical studies, inhibiting the proteases that cleave surface-bound MSLN decreased MSLN shedding^21–23^. Our PI experiments found that at 10 nM of anetumab ravtansine, cells treated with rMSLN had a higher percentage of confluence compared to T and M + T, suggesting that the PIs may have stabilized surface MSLN to some degree, thus allowing anetumab ravtansine to be more effective. While this signal is encouraging, these results are difficult to interpret knowing that M + T may have direct cytotoxicity since both cell viability and confluence decreased compared to all other groups (DMSO, rMSLN, M, and T) at baseline prior to the addition of anetumab ravtansine (Figs. 2 and 3). We theorize that the observed cytotoxicity is likely a synergistic effect due to the combination of PIs since neither have significant cytotoxicity on their own^26,27^. However, there are no studies in the literature commenting on the use of M + T in combination. As marimastat and TMI-1 have broad activity and act on cell surface proteins other than MSLN, it is possible that the combined inhibition of MMP and ADAM17 resulted in this unanticipated effect through a mechanism not related to MSLN. We also found that our 100 nM experiments had low cell counts, which may explain the lack of difference in viability and confluence, as well as the higher viability seen compared to 10 and 50 nM. The higher concentration of anetumab ravtansine significantly reduced the confluence of our model (Fig. 3), so there were fewer cells and wider confidence intervals in our measurements of viability of the remaining cells (Fig. 2).

A novel mechanism to reduce soluble oncogenic proteins is TPE as seen with sPD-L1 and PD-L1 positive EVs, which could avoid the potential for the off-target effects and toxicity from PIs^11^. Currently, there is an ongoing clinical trial studying TPE plus radiation to improve clinical outcomes in patients with melanoma refractory to ICIs (NCT04581382). To the authors’ knowledge, there are no other studies investigating ways to reduce soluble oncogenic proteins. Based on the encouraging results from the sPD-L1 study showing that TPE can reduce sPD-L1, we investigated the feasibility of using TPE to reduce sMSLN. Since sMSLN is present at low levels in the blood of patients without cancer, we included samples from any patient undergoing TPE^28^. One session of TPE decreased sMSLN in a manner similar to sPD-L1, but to a lesser degree with a 43.6% reduction on average compared to 70.8% with each TPE session for sPD-L1^11^. The sPD-L1 study had more patients (24 versus 15 in our study), which could account for the differences in soluble protein reduction. Another limitation to our study is that the plasma samples analyzed were from the original Orme et al. study, which were taken from frozen storage, and we only have results after one TPE session, rather than multiple points in time. We also note a wide variation in sMSLN reduction. TPE removes plasma-restricted substances that are too large for rapid diffusion, and it is unknown whether sMSLN is completely plasma restricted, non-diffusing, and unbound. Additionally, there may be a mix of sMSLN sizes such that TPE clears the larger proteins, but not the smaller ones. These results require prospective validation in a larger cohort and could benefit from an expanded study into the effects of multiple TPE sessions. We suggest future studies evaluate what percentage reduction in sMSLN is clinically relevant, which could be done with preclinical experiments analyzing treatment effects of MSLN-directed therapies in the presence of varying levels of sMSLN.

While MSLN-directed therapies have focused on patients with mesothelioma, if a MSLN-directed therapy was found to be effective, then it could have a broader impact for patients with other solid tumors, including lung, gynecologic, pancreatic, head and neck, and esophageal cancers^1–3^. Specifically, within pancreatic cancer, sMSLN was recently implicated in facilitating metastatic spread^29^. Metastatic pancreatic cells secrete high levels of MSLN resulting in increased accumulation of CD206 + macrophages, which was associated with worse clinical outcomes. Within those macrophages, MSLN increased levels of VEGF-A and S100 calcium binding protein A9 (S100A9) expression. S100A9 increased neutrophil infiltration and recruitment to the lungs, in addition to neutrophil extracellular trap formation. When MSLN was genetically depleted, tumor growth and metastasis were inhibited, and macrophages skewed towards a tumor suppressing phenotype^29^. There is also evidence that MSLN promotes the development of pro-tumorigenic macrophages^30^. Taken together, these studies suggest that MSLN has a key role in cancer growth and metastasis beyond mesothelioma.

Overall, our results indicate that high levels of sMSLN represent a mechanism of resistance to anti-MSLN antibody-based therapies, TMI-1 may stabilize MSLN at the cell surface, and TPE could offer a novel approach to reduce sMSLN. Future studies of MSLN-directed therapies should consider the stability of surface MSLN and the effects of sMSLN on treatment efficacy. sMSLN levels could predict which patients may benefit from MSLN-directed therapies and if patients have high sMSLN, then resistance could be mitigated with stabilization of surface MSLN or reducing sMSLN. Going forward, we foresee clinical trials using sMSLN levels to stratify patients. If patients have high sMSLN levels, then they could undergo pre-treatment with TPE, or another modality to reduce sMSLN, prior to receiving the MSLN-directed therapy to optimize response. Patients with low sMSLN may benefit from going directly to the therapeutic option being studied. The success of current and future clinical trials investigating MSLN therapies may rely on incorporating sMSLN as a predictive biomarker.

## Methods

### Therapeutic plasma exchange

Patients were prospectively enrolled after obtaining written or signed informed consent as a part of Orme et al.’s^11^ study between December 2019 and March 2020. Whole blood samples were collected before and after a one plasma volume of TPE with albumin as the replacement fluid in patients undergoing routine TPE for various medical conditions, such as autoimmune diseases and hyperviscosity syndromes (Table 1). The first 8 mL was discarded to avoid contamination. Plasma was separated from whole blood by centrifugation. Plasma waste samples from the procedure itself was also collected. Anticoagulation related to the procedure was acid citrate dextrose solution A (ACD-A) with or without unfractionated heparin.

sMSLN levels were measured with an ELISA assay (RayBiotech, Norcross GA, USA) in matched pre- and post-TPE plasma samples. Plasma samples were diluted 1:10 before applying to the plate. The Wilcoxon signed rank exact test was used to compare sMSLN between pre and post plasma samples.

### Anetumab immunoprecipitation

The anti-MSLN antibody anetumab (Invitrogen, Waltham MA, USA) was covalently coupled to Dynabeads® (Life Technologies, Waltham MA, USA) according to manufacturer instructions at 5 µg per mg of beads. Three effective concentrations (12.5 µg/mL, 25 µg/ml, 37.5 µg/mL) of anetumab-conjugated Dynabeads® were used to immunoprecipitate MSLN from two randomly selected plasma samples from the pre-TPE blood draws. Assays were performed in duplicate. The Wilcoxon signed-rank test was used to compare MSLN levels before and after immunoprecipitation.

### Protease inhibitors and cytotoxicity

The epithelioid mesothelioma cell line Mero-95 was grown in RPMI-1640 supplemented with 10% FBS, 100 U/mL penicillin and 100 µg/mL streptomycin. For each experiment, cells were plated at a seeding density of 10,000 cells/well for 24 h before adding protease inhibitors. Protease inhibitors were diluted in growth medium and added to each well, using 1% DMSO as a vehicle control, and allowed to incubate for 20 h. Then, wells were washed with PBS before anetumab ravtansine (Selleck Chemicals, Houston TX, USA) was added at concentrations of 10 nM, 50 nM, or 100 nM, and incubated for 48 h. To determine the effects of sMSLN on cytotoxicity, anetumab ravtansine was pre-incubated with recombinant MSLN (rMSLN, Creative Biomart, Shirley NY, USA) prior to being added to Mero-95 cells. For each anetumab ravtansine concentration, we had groups with (1) no pre-treatment, (2) 1% DMSO for a control (3) recombinant MSLN (rMSLN, 3 nM) as a surrogate for sMSLN, (4) marimastat (M) (Selleck Chemicals, Houston TX, USA) 10 µM, (5) TMI-1 (T) (Bio-techne, Minneapolis MN, USA) 10 µM, (6) combined marimastat and TMI-1 (M + T), each at 10 µM. After 48 h, we conducted a WST-8/1-methoxy PMS (Selleck Chemicals, Houston TX, USA) cytotoxicity assay to determine viability. For the WST-8 assay, WST-8 at a final concentration of 0.5 mM was added with 1-methoxy PMS at a final concentration of 20 μM and incubated in the cell culture chamber for two hours. Results were read on a Glomax Explorer plate reader at 450 nm with a reference wavelength at 650 nm. DMSO at a 1% final concentration was used as a positive control, applied to cells at the same time as the protease inhibitors. Groups were compared with a 2-way ANOVA and a *p* value less than 0.05 was considered significant. Graphpad Prism was used to perform Ordinary Two-way ANOVA with Tukey’s multiple comparisons test to analyze the viability and confluence results.

## Electronic supplementary material

Below is the link to the electronic supplementary material.

Supplementary Material 1

## Data Availability

All data generated or analyzed during this study are included in this published article (and its Supplementary Information files).
