# Supplementary material for: Evaluating therapeutic plasma exchange and protease inhibitors as mechanisms to reduce soluble mesothelin
Source: Sci Rep. 2025 Apr 16;15:13174. doi: 10.1038/s41598-025-97952-x (PMC12003639; doi:10.1038/s41598-025-97952-x)
Supplement: Supplementary file 1 — Supplementary Material 1 [file 41598_2025_97952_MOESM1_ESM.pdf]

Supplementary Table 1. Viability Results.

	**Predicted (LS) mean diff.**	**95.00% CI of diff.**	**Adjusted P Value**
**PBS control**			
No pre-Tx vs. rMSLN	12.05	-23.62 to 47.72	0.8946
No pre-Tx vs. DMSO	-4.75	-40.42 to 30.92	0.9982
No pre-Tx vs. M	5.15	-30.52 to 40.82	0.9974
No pre-Tx vs. T	5.95	-29.72 to 41.62	0.9948
No pre-Tx vs. M+T	48.25	12.58 to 83.92	0.0042
rMSLN vs. DMSO	-16.8	-52.47 to 18.87	0.6873
rMSLN vs. M	-6.9	-42.57 to 28.77	0.9897
rMSLN vs. T	-6.1	-41.77 to 29.57	0.9942
rMSLN vs. M+T	36.2	0.5326 to 71.87	0.0453
DMSO vs. M	9.9	-25.77 to 45.57	0.951
DMSO vs. T	10.7	-24.97 to 46.37	0.9331
DMSO vs. M+T	53	17.33 to 88.67	**0.0016**
M vs. T	0.8	-34.87 to 36.47	>0.9999
M vs. M+T	43.1	7.433 to 78.77	**0.012**
T vs. M+T	42.3	6.633 to 77.97	**0.014**
**10nM ARav**			
No pre-Tx vs. rMSLN	-9.5	-45.17 to 26.17	0.9587
No pre-Tx vs. DMSO	3.9	-39.78 to 47.58	0.9997

No pre-Tx vs. M	8.05	-27.62 to 43.72	0.9796
No pre-Tx vs. T	12.7	-22.97 to 48.37	0.8724
No pre-Tx vs. M+T	18.8	-16.87 to 54.47	0.5813
rMSLN vs. DMSO	13.4	-30.28 to 57.08	0.927
rMSLN vs. M	17.55	-18.12 to 53.22	0.648
rMSLN vs. T	22.2	-13.47 to 57.87	0.4065
rMSLN vs. M+T	28.3	-7.367 to 63.97	0.1757
DMSO vs. M	4.15	-39.53 to 47.83	0.9996
DMSO vs. T	8.8	-34.88 to 52.48	0.9877
DMSO vs. M+T	14.9	-28.78 to 58.58	0.8908
M vs. T	4.65	-31.02 to 40.32	0.9984
M vs. M+T	10.75	-24.92 to 46.42	0.9319
T vs. M+T	6.1	-29.57 to 41.77	0.9942
**50nM ARav**			
No pre-Tx vs. rMSLN	-2.15	-37.82 to 33.52	>0.9999
No pre-Tx vs. DMSO	1.35	-34.32 to 37.02	>0.9999
No pre-Tx vs. M	-5.85	-41.52 to 29.82	0.9952
No pre-Tx vs. T	-5.1	-40.77 to 30.57	0.9975
No pre-Tx vs. M+T	7.9	-27.77 to 43.57	0.9812
rMSLN vs. DMSO	3.5	-32.17 to 39.17	0.9996
rMSLN vs. M	-3.7	-39.37 to 31.97	0.9995

rMSLN vs. T	-2.95	-38.62 to 32.72	0.9998
rMSLN vs. M+T	10.05	-25.62 to 45.72	0.9479
DMSO vs. M	-7.2	-42.87 to 28.47	0.9876
DMSO vs. T	-6.45	-42.12 to 29.22	0.9924
DMSO vs. M+T	6.55	-29.12 to 42.22	0.9919
M vs. T	0.75	-34.92 to 36.42	>0.9999
M vs. M+T	13.75	-21.92 to 49.42	0.8317
T vs. M+T	13	-22.67 to 48.67	0.8613
**100nM ARav**			
No pre-Tx vs. rMSLN	24.6	-11.07 to 60.27	0.3003
No pre-Tx vs. DMSO	24.25	-19.43 to 67.93	0.5281
No pre-Tx vs. M	19.05	-16.62 to 54.72	0.568
No pre-Tx vs. T	15.2	-20.47 to 50.87	0.7671
No pre-Tx vs. M+T	39.15	3.483 to 74.82	**0.026**
rMSLN vs. DMSO	-0.35	-44.03 to 43.33	>0.9999
rMSLN vs. M	-5.55	-41.22 to 30.12	0.9962
rMSLN vs. T	-9.4	-45.07 to 26.27	0.9604
rMSLN vs. M+T	14.55	-21.12 to 50.22	0.7972
DMSO vs. M	-5.2	-48.88 to 38.48	0.9989
DMSO vs. T	-9.05	-52.73 to 34.63	0.986
DMSO vs. M+T	14.9	-28.78 to 58.58	0.8908

M vs. T	-3.85	-39.52 to 31.82	0.9993
M vs. M+T	20.1	-15.57 to 55.77	0.5123
T vs. M+T	23.95	-11.72 to 59.62	0.3272
ARav = anetumab ravtansine; Tx = treatment; rMSLN = recombinant mesothelin; M = marimastat; T = TMI-1; M+T = combined marimastat and TMI-1			

Supplementary Table 2. Confluence Results.

**PBS control**	**Mean Diff.**	**95.00% CI of diff.**	**Adjusted P Value**
No pre-Tx vs. rMSLN	0.9	-17.33 to 19.13	>0.9999
No pre-Tx vs. DMSO	13.94	-4.292 to 32.17	0.2085
No pre-Tx vs. M	6.18	-12.05 to 24.41	0.8966
No pre-Tx vs. T	11.55	-6.682 to 29.78	0.3934
No pre-Tx vs. M+T	31.61	13.38 to 49.84	**0.0002**
rMSLN vs. DMSO	13.04	-5.192 to 31.27	0.2692
rMSLN vs. M	5.28	-12.95 to 23.51	0.9439
rMSLN vs. T	10.65	-7.582 to 28.88	0.4807
rMSLN vs. M+T	30.71	12.48 to 48.94	**0.0003**
DMSO vs. M	-7.76	-25.99 to 10.47	0.7736
DMSO vs. T	-2.39	-20.62 to 15.84	0.9984
DMSO vs. M+T	17.67	-0.5618 to 35.90	0.0613
M vs. T	5.37	-12.86 to 23.60	0.9399
M vs. M+T	25.43	7.198 to 43.66	**0.0029**

T vs. M+T	20.06	1.828 to 38.29	**0.0252**
**10nM ARav**			
No pre-Tx vs. rMSLN	-12.45	-30.68 to 5.782	0.315
No pre-Tx vs. DMSO	6.76	-11.47 to 24.99	0.857
No pre-Tx vs. M	4.93	-13.30 to 23.16	0.9576
No pre-Tx vs. T	7.25	-10.98 to 25.48	0.8183
No pre-Tx vs. M+T	8.13	-10.10 to 26.36	0.7388
rMSLN vs. DMSO	19.21	0.9782 to 37.44	0.0348
rMSLN vs. M	17.38	-0.8518 to 35.61	0.0679
rMSLN vs. T	19.7	1.468 to 37.93	**0.0289**
rMSLN vs. M+T	20.58	2.348 to 38.81	**0.0206**
DMSO vs. M	-1.83	-20.06 to 16.40	0.9996
DMSO vs. T	0.49	-17.74 to 18.72	>0.9999
DMSO vs. M+T	1.37	-16.86 to 19.60	0.9999
M vs. T	2.32	-15.91 to 20.55	0.9986
M vs. M+T	3.2	-15.03 to 21.43	0.9937
T vs. M+T	0.88	-17.35 to 19.11	>0.9999
**50nM ARav**			
No pre-Tx vs. rMSLN	-7.54	-25.77 to 10.69	0.7933
No pre-Tx vs. DMSO	-1.32	-19.55 to 16.91	>0.9999
No pre-Tx vs. M	-5.15	-23.38 to 13.08	0.9493

No pre-Tx vs. T	-4.4	-22.63 to 13.83	0.9738
No pre-Tx vs. M+T	1.59	-16.64 to 19.82	0.9998
rMSLN vs. DMSO	6.22	-12.01 to 24.45	0.8941
rMSLN vs. M	2.39	-15.84 to 20.62	0.9984
rMSLN vs. T	3.14	-15.09 to 21.37	0.9942
rMSLN vs. M+T	9.13	-9.102 to 27.36	0.6381
DMSO vs. M	-3.83	-22.06 to 14.40	0.9857
DMSO vs. T	-3.08	-21.31 to 15.15	0.9947
DMSO vs. M+T	2.91	-15.32 to 21.14	0.996
M vs. T	0.75	-17.48 to 18.98	>0.9999
M vs. M+T	6.74	-11.49 to 24.97	0.8584
T vs. M+T	5.99	-12.24 to 24.22	0.9081
**100nM ARav**			
No pre-Tx vs. rMSLN	0.06	-18.17 to 18.29	>0.9999
No pre-Tx vs. DMSO	1.11	-17.12 to 19.34	>0.9999
No pre-Tx vs. M	1.06	-17.17 to 19.29	>0.9999
No pre-Tx vs. T	0	-18.23 to 18.23	>0.9999
No pre-Tx vs. M+T	4.65	-13.58 to 22.88	0.9668
rMSLN vs. DMSO	1.05	-17.18 to 19.28	>0.9999
rMSLN vs. M	1	-17.23 to 19.23	>0.9999
rMSLN vs. T	-0.06	-18.29 to 18.17	>0.9999

rMSLN vs. M+T	4.59	-13.64 to 22.82	0.9686
DMSO vs. M	-0.05	-18.28 to 18.18	>0.9999
DMSO vs. T	-1.11	-19.34 to 17.12	>0.9999
DMSO vs. M+T	3.54	-14.69 to 21.77	0.99
M vs. T	-1.06	-19.29 to 17.17	>0.9999
M vs. M+T	3.59	-14.64 to 21.82	0.9893
T vs. M+T	4.65	-13.58 to 22.88	0.9668
ARav = anetumab ravtansine; Tx = treatment; rMSLN = recombinant mesothelin; M = marimastat; T = TMI-1; M+T = combined marimastat and TMI-1			

Supplementary Figure 1.

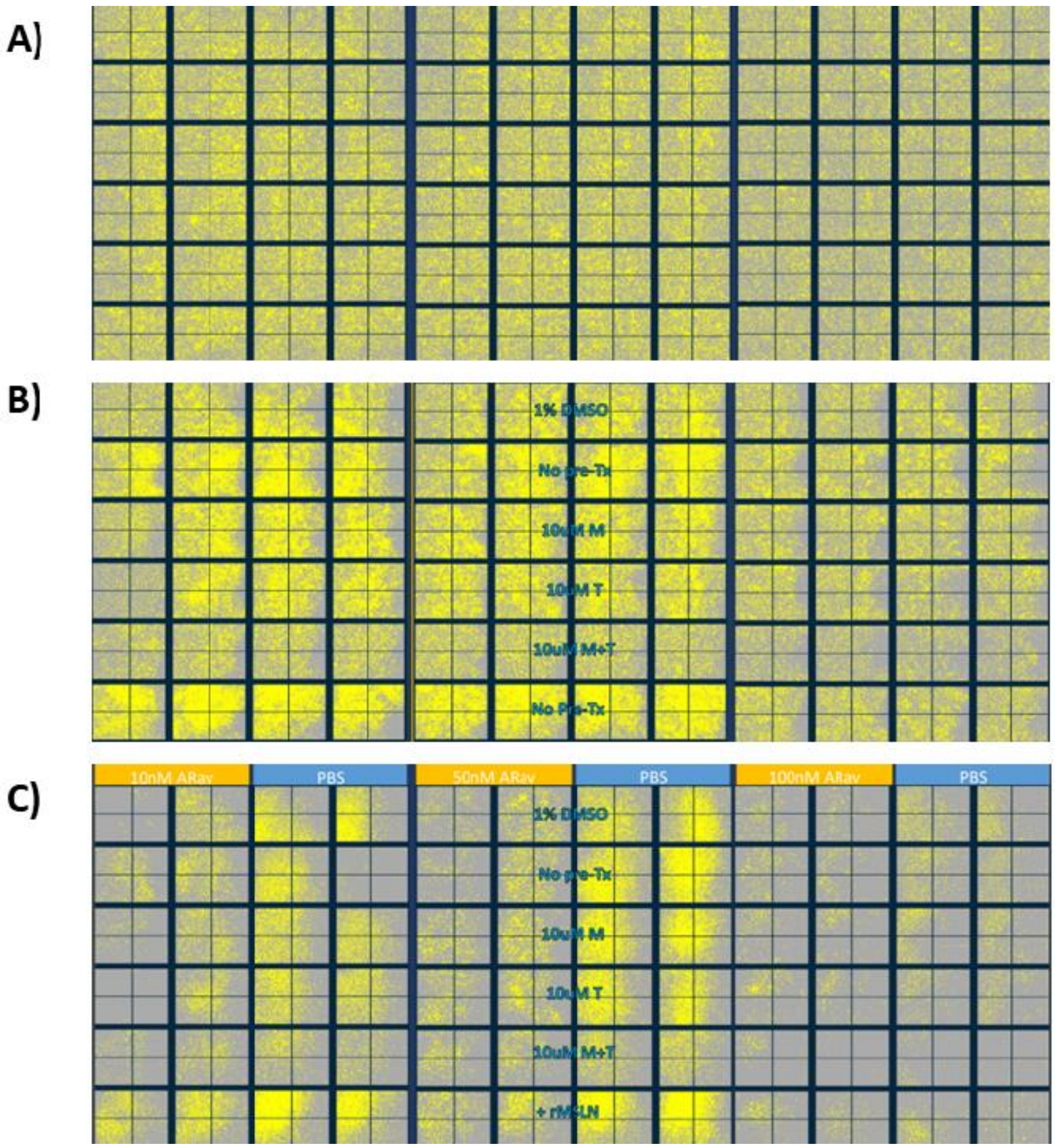

Confluence Images. A) Before any treatments approximately 20 hours after plating 10,000 cells/well. B) After 20 hours of protease inhibitor pretreatment, prior to adding anetumab ravtansine (ARav) or PBS. From top to bottom: 1% DMSO, no pre-treatment (pre-tx), 10 μM marimastat (M), 10 μM TMI-1 (T), 10 μM of combined marimastat and TMI-1 (M+T), no pre-

tx. C) 48 hours after the addition of ARav or PBS. Top to bottom: 1% DMSO, no pre-treatment (pre-tx), 10 μM marimastat (M), 10 μM TMI-1 (T), 10 μM of combined marimastat and TMI-1 (M+T), recombinant mesothelin (rMSLN). Left to right: 10 nM ARav, PBS, 50 nM ARav, PBS, 100 nM ARav, PBS.
